# Spherical-Cap Approximation of Vector Quantization for Quantization-Based Combining in MIMO Broadcast Channels with Limited Feedback

**DOI:** 10.3390/s22145146

**Published:** 2022-07-08

**Authors:** Moonsik Min, Tae-Kyoung Kim

**Affiliations:** 1 School of Electronics Engineering, Kyungpook National University, Daegu 41566, Korea; msmin@knu.ac.kr; 2 School of Electronic and Electrical Engineering, Kyungpook National University, Daegu 41566, Korea; 3 Department of Electronic Engineering, Gachon University, Seongnam 13120, Korea

**Keywords:** multiple-input multiple-output (MIMO), channel state information, vector quantization, limited feedback, quantization-based combining

## Abstract

The spherical-cap approximation of vector quantization (SCVQ) is an analytical model used for the mathematical analysis of multiple-input multiple-output (MIMO) systems with limited feedback. SCVQ closely emulates the distribution of the quantization error induced by the finite-rate quantization of a channel using a simple and analytically tractable approach. However, the conventional SCVQ model is not applicable when antenna-combining schemes such as quantization-based combining (QBC) are considered. Because QBC is an effective antenna-combining method that minimizes channel quantization errors, it can be adopted for various practical MIMO broadcast systems. Nevertheless, evaluating the performance of QBC-based MIMO systems with an explicit codebook can be extremely difficult, depending on the system complexity. To resolve this, this study generalizes the conventional SCVQ to be compatible with the QBC. The proposed generalized version of the SCVQ effectively emulates the quantization error obtained using QBC, while enabling a simple simulation independent of the number of feedback bits and mathematically tractable analysis. We validate the effectiveness of the proposed model by presenting a wireless communication application based on a dense cellular network.

## 1. Introduction

Multiple-input multiple-output (MIMO) systems implement multiple antennas at both the transmitter and receiver to exploit additional wireless communication resources along spatial layers [1,2]. MIMO systems achieve a linear increase in capacity, which is called spatial multiplexing gain. Because of this advantage, various versions of MIMO systems have been extensively studied as promising technologies for evolving wireless communication [3,4,5,6]. In MIMO systems, the amount of channel state information (CSI) at the transmitter significantly affects the downlink capacity. In practice, realizing an accurate CSI at the transmitter (CSIT) may be challenging, particularly for frequency-division duplex (FDD)-based systems, because the transmitter in the FDD systems cannot directly track its downlink channel. Thus, in MIMO downlink systems with FDD, a form of CSI should be reported from the receiver to the transmitter, where the receiver can estimate the CSI using a pilot. Limited feedback is a conventional example of CSI feedback in which the receiver quantizes the CSI using a codebook and feeds the quantized CSI back to the transmitter [7,8,9,10,11,12,13,14,15].

To apply the limited feedback method, the transmitter and receiver share a predefined codebook to obtain a quantized CSIT. In practice, the codebook must be fixed beforehand and known to both the transmitter and receiver such that the transmitter can find the codeword that approximates the true downlink channel using the codebook index fed back from the receiver [7,8]. However, with this type of fixed codebook, mathematical analysis of MIMO systems can be extremely difficult, depending on their complexity. To resolve this, random vector quantization (RVQ) is considered as a lower-bound approximation in terms of the codebook construction [9]. However, as MIMO systems in cellular networks become complicated, RVQ-based performance evaluation becomes difficult, particularly when system-level performance evaluation is conducted [12,16]. As an alternative solution, an upper-bound approximation was introduced in which the cumulative distribution function (CDF) of the quantization error of vector quantization was approximated by adopting an upper bound on the quantization cell [12,16]; this is referred to as spherical-cap approximation of vector quantization (SCVQ), as presented in [16]. SCVQ provides a simple analytical model that closely approximates a realistic channel quantization process that uses an explicit codebook. Therefore, it has been widely used to analyze limited-feedback-based MIMO systems [12,16,17].

### 1.1. Related Work

Extensive research on codebook-based limited (or finite-rate) feedback has been conducted over the past few decades to enable spatial division multiplexing (SDM), particularly when the transmitter cannot directly track the downlink channels. Early work in the literature typically used RVQ for codebook design to enable the mathematical analysis of the downlink achievable rate. In [2], the ergodic downlink rate of multiuser MIMO broadcast channels with finite-rate channel feedback was analyzed assuming that (1) zero-forcing beamforming (ZFBF) is used to simultaneously serve multiple users, (2) the number of users is equal to the number of transmit antennas, (3) the downlink channels of all users are independent and have only a small-scale fading effect and (4) the number of receive antennas of each user is 1. Based on the RVQ, the results in [2] are extended to the case where each user has multiple receive antennas, and they are used to minimize the quantization error [9]. Instead of using an explicit codebook such as RVQ, quantization cell approximations, referred to as SCVQ in this paper, were proposed to derive a universal lower bound on the outage probability for any finite set of beamformers [15], and to derive an SER lower bound that is tight for good beamformer designs [18]. The SCVQ was invented mainly for mathematical tractability when performance analysis was extremely difficult with an explicit codebook including RVQ. Explicit codebook-based analysis is more difficult for more complicated systems. For example, consider a network in which the number of users is much greater than the number of transmit antennas. Selecting appropriate users can significantly increase the downlink sum rate in this case. To mathematically evaluate the increasing rate, the authors in [5] derived the CDF of the signal-to-noise-plus-interference ratio (SINR) using SCVQ and applied the extreme value theory to derive the asymptotic increasing rate of the downlink sum capacity with respect to the number of users. It should be noted that the corresponding analysis is extremely difficult if we consider an explicit codebook, rather than using the SCVQ model. If we further consider a multicell network, the analysis becomes more complicated such that the SCVQ is preferred for system-level performance analysis. Based on SCVQ, the capacity was analyzed when a multiantenna transmission was used in a random ad hoc network in which the locations of access points were distributed based on the Poisson point process (PPP) [19]. The performance of a MIMO system with limited feedback in a random cellular network was analyzed in [16,20,21]. The ergodic secrecy rate was analyzed in multiantenna cellular systems with limited feedback in [22]. In Table 1, the previous studies introduced in this study are classified depending on the quantization model used for limited feedback. In addition, the fundamental properties of the RVQ and SCVQ are also compared.

However, the SCVQ used in the studies so far is applicable only when the number of receive antennas is restricted to one, because it is derived by assuming a vector channel between the transmitter and receiver, i.e., derived for multiple-input and single-output channels. In [23], the asymptotic performance of the SCVQ was verified, and an extended version of the SCVQ, which was derived assuming multiple receive antennas, was proposed. However, the extended SCVQ in [23] was derived assuming a full-stream SDM for each user and was validated only when a suboptimal quantization criterion was used. To the best of the authors’ knowledge, an SCVQ that is generally applicable when receive antenna combining is used to improve the quantization performance does not exist. Thus, if we use an antenna-combining method associated with the quantization process, such as quantization-based combining (QBC) [10], conventional SCVQ will not provide a close approximation. QBC is an effective antenna-combining method that reduces the quantization error caused by limited feedback using multiple receive antennas, and it has been widely employed to enhance feedback performance [24,25,26,27]. Thus, if we have an appropriate analytical model, such as SCVQ appropriate for QBC, it can be effectively used to evaluate the performance of various complicated MIMO systems utilizing QBC. Deriving an SCVQ model compatible with QBC can dramatically simplify the theoretical analysis of multiple-antenna systems based on limited feedback with QBC, as was done in previous studies mentioned in this subsection, such that the effect of multiple receive antennas can be mathematically analyzed to provide insight into the advantages and disadvantages of using receive antennas to reduce the quantization error in a complicated wireless network.

### 1.2. Contribution

In this study, a generalized SCVQ model compatible with QBC is proposed. The proposed model provides an upper-bound approximation for the vector quantization problem of the effective channel when QBC is used at the receiver end to reduce the quantization error. Specifically, the upper bound for the CDF of the quantization error is derived and used to obtain a random variable that closely approximates the original quantization error in the probability distribution. Therefore, conducting simulations and mathematical analysis to evaluate MIMO systems with QBC using the proposed approximation is significantly easier. To verify this, we consider a dense cellular network as a wireless communication application, in which each base station (BS) performs zero-forcing beamforming with QBC-based limited feedback to simultaneously serve multiple users. In this network, we evaluate the optimal number of feedback bits as a function of the channel coherence time. As the optimal number is considerably large, simulating the conventional vector quantization process with an explicit codebook requires significant computation time owing to the massive codebook size. In contrast, the simulation complexity of the proposed method does not depend on the number of feedback bits; thus, evaluating the optimal number is comparatively easy. Moreover, performance analysis becomes mathematically tractable using the proposed SCVQ, which is significantly difficult using conventional codebook-based quantization. This advantage is demonstrated using a single-user example.

The remainder of this paper is organized as follows. Section 2 describes the system model and preliminaries, Section 3 derives the proposed SCVQ model, Section 4 presents a wireless communication application and the corresponding simulation results to verify the validity of the proposed model, and Section 5 draws the conclusions.

Notation: Matrices are denoted with upper-case boldface letters, and column vectors are denoted with lower-case boldface letters. The superscripts ${\left(\cdot \right)}^{T}$ and ${\left(\cdot \right)}^{H}$ indicate the transpose and complex conjugate transpose of a matrix, respectively. $I_{m}$ denotes the m×m identity matrix. ∥·∥ indicates the vector norm and |·| is the absolute value of a complex number. Pr(·) denotes the probability of an event and E(·) denotes the expectation. Finally, N, R and C represent the sets of natural, real and complex numbers, respectively.

## 2. System Model and Preliminaries

In discrete-time wireless communication systems, a MIMO channel between a transmitter and receiver is commonly modeled by an $N_{t}\times N_{r}$ complex random matrix H, where $N_{t}$ and $N_{r}$ represent the numbers of the transmit and receive antennas, respectively. Let x be the transmit signal vector radiating from $N_{t}$ transmit antennas. Then, the signal received at $N_{r}$ receive antennas can be represented as

(1) $$\begin{array}{cc} \; & y={\left[y_{1},\cdots ,y_{N_{r}}\right]}^{T}=H^{H}x+z, \end{array}$$

where the *i*-th element of $z\in C^{N_{r}\times 1}$ denotes the zero-mean complex additive white Gaussian noise at the *i*-th receive antenna. In this study, the variance of each element of z is normalized to 1. In FDD downlink systems, the transmitter or BS cannot directly track the downlink channel. Thus, the BS requires CSI feedback from the receiver to perform channel-dependent signal processing at the transmitter end. Limited feedback is a typical method in which each user estimates, quantizes and feeds back the CSI to the transmitter using a finite number of quantization (or feedback) bits. QBC is a receiving antenna-combining technique that effectively reduces quantization errors in the limited feedback method [10].

### 2.1. Limited Feedback

In this study, receive antenna combining refers to linearly combining received signals $y_{1},\cdots ,y_{N_{r}}$ using a unit receive combining vector, $\gamma \in C^{N_{r}\times 1},∥\gamma ∥=1,$ to convert the MIMO channel H into a vector channel; we denote the resulting vector channel by the effective channel $h^{e}$ such that $h^{e}=H\gamma \in C^{N_{t}\times 1}$. We assume perfect channel estimation at the receiver end, which is a typical assumption when the effect of channel quantization is mainly considered [2,9,10]. To apply the limited feedback method, the receiver quantizes the channel direction information (CDI) using a predefined codebook $C=\left\{c_{1},\ldots ,c_{2^{B}}\right\}$, which is previously fixed and known to the serving BS; *B* denotes the number of feedback bits per user. Because the receiver quantizes the direction of the effective channel $h^{e}$, each codeword is a unit vector in $C^{N_{t}\times 1}$, which differs from all the other codewords. The quantized CDI is expressed as

(2) $$\begin{array}{c} \hat{h}=\underset{c\in C}{\mathrm{argmin}}d\left(c,h^{e}\right), \end{array}$$

where d(·,·) is a distance measure that depends on the quantization strategy (we discuss it further in the following section). The codeword index corresponding to $\hat{h}$ in C is reported to the transmitter, and the transmitter can obtain the quantized CDI from the codebook.

Because the number of quantization bits is finite, the performance is limited by the quantization error, which is characterized as

(3) $$\begin{array}{c} \sin^{2}\theta =1-{\left|{\hat{h}}^{H}\tilde{h}\right|}^{2},\;\;\tilde{h}=\frac{h^{e}}{∥h^{e}∥}, \end{array}$$

where $\theta \in [0,\frac{\pi }{2}]$ is the angle between $\hat{h}$ and $\tilde{h}$. Subsequently, the original CDI can be represented as

(4) $$\begin{array}{cc} \tilde{h}\; & =\hat{h}e^{\phi \sqrt{-1}}\cos\theta +g\sin\theta , \end{array}$$

where the unit vector g lies in the left null-space of $\hat{h}$ and ϕ satisfies $e^{\phi \sqrt{-1}}=\frac{{\hat{h}}^{H}\tilde{h}}{|{\hat{h}}^{H}\tilde{h}|}$.

When $N_{r}=1$, i.e., in multiple-input and single-output downlink channels, the chordal distance between the unit vectors is typically used as a distance measure for quantization in (2) [8,9,12]. In this case, the CDF of the quantization error $\sin^{2}\theta$ is derived as [9]

(5) $$\begin{array}{c} F_{1}\left(x\right)=1-{\left(1-x^{N_{t}-1}\right)}^{2^{B}}, \end{array}$$

assuming an RVQ. As communication systems have become more complex, conducting a theoretical analysis of such systems using the limited feedback method has become increasingly difficult. As an alternative solution, the following upper-bound approximation of the CDF of $\sin^{2}\theta$ was proposed [12]:

(6) $$\begin{array}{c} F_{1}\left(x\right)\approx \left\{\begin{array}{cc} 2^{B}x^{N_{t}-1}, & 0\leq x\leq \delta_{1}\\ 1, & x>\delta_{1} \end{array}\right., \end{array}$$

where $\delta_{1}=2^{-\frac{B}{N_{t}-1}}$. With this approximation, $\sin^{2}\theta$ can be equivalently represented by a beta random variable multiplied by $\delta_{1}$; thus, the theoretical analysis of the SINR or the corresponding ergodic capacity becomes considerably easier. This approximation is called SCVQ [16] and provides a close approximation, particularly when *B* is sufficiently large. However, the conventional SCVQ in (5) is valid only when $N_{r}=1$; thus, it is not applicable when receive antenna combining is used.

### 2.2. Quantization-Based Combining

The QBC determines the receive combining vector γ such that the quantization error $\sin^{2}\theta$ between the effective and quantized CDI is minimized. The QBC procedure is summarized as follows [10]:

1. Find an orthonormal basis $\left\{q_{1},\cdots ,q_{N_{r}}\right\}$ that spans the column space of H.
2. The quantized CDI is determined as the closest vector in C to the column space of H:
  (7) $$\begin{array}{c} \hat{h}=\underset{c\in C}{\mathrm{argmax}}∥Q^{H}{c∥}^{2}=\underset{c\in C}{\mathrm{argmin}}[1-∥Q^{H}c{∥}^{2}],\;\;Q=\left[q_{1},\cdots ,q_{N_{r}}\right]. \end{array}$$
3. Determine the direction of the effective channel by projecting $\hat{h}$ onto the column space of H:
  (8) $$\begin{array}{c} s^{\mathrm{proj}}=\frac{QQ^{H}\hat{h}}{∥QQ^{H}\hat{h}∥}. \end{array}$$
4. The receive combining weight vector is finally detemined as
  (9) $$\begin{array}{c} \gamma =\frac{{\left(H^{H}H\right)}^{-1}H^{H}s^{\mathrm{proj}}}{∥{\left(H^{H}H\right)}^{-1}H^{H}s^{\mathrm{proj}}∥}. \end{array}$$

## 3. SCVQ for QBC

SCVQ is an analytical model that linearly approximates the probability distribution of the quantization error $\sin^{2}\theta$. By assuming limited feedback, the SCVQ has been widely used to simplify mathematical analysis [12,16,17,20]. SCVQ is known to provide an upper-bound performance with respect to the codebook construction, which is tight, particularly when *B* is large; thus, it is widely used to analyze the performance of limited-feedback-based MIMO systems.

Here, we generalize the conventional SCVQ as compatible with QBC. Subsequently, we demonstrate the effectiveness of the proposed SCVQ by considering a wireless communication application. In [10], it was shown that $\left(1-∥Q^{H}c{∥}^{2}\right)$ follows a beta distribution with parameters $N_{t}-N_{r}$ and $N_{r}$, for an arbitrarily chosen c∈C. Because the QBC procedure selects the argument of the minimum of $1-∥Q^{H}{c∥}^{2}$ in C as the quantized CDI, as expressed in (7), the quantization error $\sin^{2}\theta =1-{\left|{\hat{h}}^{H}\tilde{h}\right|}^{2}$ is the minimum of $2^{B}$ independent and identically distributed (i.i.d.) beta random variables with parameters $N_{t}-N_{r}$ and $N_{r}$ (Lemma 1 in [10]). Moreover, the CDF of the beta random variable with parameters $N_{t}-N_{r}$ and $N_{r}$ can be expressed as follows [28]:

(10) $$\begin{array}{c} F_{b}\left(x\right)=\sum_{i=1}^{N_{r}}\frac{\Gamma (N_{t}-N_{r}+i-1)}{\Gamma \left(N_{t}-N_{r}\right)\Gamma \left(i\right)}x^{N_{t}-N_{r}}{\left(1-x\right)}^{i-1}. \end{array}$$

Because $\sin^{2}\theta$ is the minimum of $2^{B}$ i.i.d. random samples following $F_{b}\left(x\right)$, the CDF of $\sin^{2}\theta$ can be calculated as

(11) $$\begin{array}{cc} F_{\sin^{2}\theta }\left(x\right) & =\mathrm{Pr}\left(\min[X_{1},\cdots ,X_{2^{B}}]\leq x\right)\\ \; & =\mathrm{Pr}\left(\left\{X_{1}\leq x\right\}\cup \left\{X_{2}\leq x\right\}\cup \cdots \cup \left\{X_{2^{B}}\leq x\right\}\right)\\ \; & \overset{\left(a\right)}{\leq }\mathrm{Pr}\left(X_{1}\leq x\right)+\mathrm{Pr}\left(X_{2}\leq x\right)+\cdots +\mathrm{Pr}\left(X_{2^{B}}\leq x\right)\\ \; & =2^{B}\sum_{i=1}^{N_{r}}\frac{\Gamma (N_{t}-N_{r}+i-1)}{\Gamma \left(N_{t}-N_{r}\right)\Gamma \left(i\right)}x^{N_{t}-N_{r}}{\left(1-x\right)}^{i-1}\\ \; & \overset{\left(b\right)}{\leq }2^{B}\left(\sum_{i=1}^{N_{r}}\frac{\Gamma (N_{t}-N_{r}+i-1)}{\Gamma \left(N_{t}-N_{r}\right)\Gamma \left(i\right)}\right)x^{N_{t}-N_{r}}, \end{array}$$

where (a) follows from the union bound on the probability of a union and (b) follows because $0\leq 1-\sin^{2}\theta \leq 1$. Consequently, the following spherical-cap approximation appropriate for the QBC can be obtained:

(12) $$\begin{array}{c} F_{\sin^{2}\theta }\left(x\right)\approx F_{\sin^{2}\theta }^{\mathrm{SCVQ}}\left(x\right)≜\left\{\begin{array}{cc} 2^{B}\left(\sum_{i=1}^{N_{r}}\frac{\Gamma (N_{t}-N_{r}+i-1)}{\Gamma \left(N_{t}-N_{r}\right)\Gamma \left(i\right)}\right)x^{N_{t}-N_{r}}, & 0\leq x\leq \delta^{*}\\ 1, & x>\delta^{*} \end{array}\right., \end{array}$$

where

(13) $$\begin{array}{c} \delta^{*}={\left(2^{B}\sum_{i=1}^{N_{r}}\frac{\Gamma (N_{t}-N_{r}+i-1)}{\Gamma \left(N_{t}-N_{r}\right)\Gamma \left(i\right)}\right)}^{-\frac{1}{N_{t}-N_{r}}}. \end{array}$$

Using (12), the quantization error $\sin^{2}\theta$, which is a random variable, can be probabilistically approximated as

(14) $$\begin{array}{c} \sin^{2}\theta \overset{d}{\approx }\delta^{*}\cdot \beta_{N_{t}-N_{r},1}, \end{array}$$

with an arbitrarily chosen beta random variable $\beta_{N_{t}-N_{r},1}$ with parameters $N_{t}-N_{r}$ and 1, where $\overset{d}{\approx }$ denotes the approximation in distribution. Consequently, both simulation and mathematical analysis can be conducted to evaluate recent communication systems with limited feedback, using this simplified approximation of the quantization error (14).

Meanwhile, the CDF of the beta random variable in (10) can be used to calculate the exact CDF of $\sin^{2}\theta$ as follows:

(15) $$\begin{array}{c} F_{\sin^{2}\theta }\left(x\right)=1-{\left(1-\sum_{i=1}^{N_{r}}\frac{\Gamma (N_{t}-N_{r}+i-1)}{\Gamma \left(N_{t}-N_{r}\right)\Gamma \left(i\right)}x^{N_{t}-N_{r}}{\left(1-x\right)}^{i-1}\right)}^{2^{B}}, \end{array}$$

which requires the application of the minimum-order statistic of i.i.d. random variables [29]. However, this exact CDF has a mathematically intractable form, which is difficult to use for the sophisticated mathematical analysis of complicated MIMO systems.

## 4. Communication Application and Simulation Results

In this section, a downlink cellular network with limited feedback is considered as a wireless communication application to verify the proposed generalized SCVQ. We examine the optimal number of feedback bits based on simulations and simple mathematical analysis. In the network, BSs are randomly distributed according to a random point process. Within the Voronoi region of each BS, *K* users are randomly located, where the user locations are independent of the BS locations. Each user is served by the nearest BS, i.e., *K* users in the Voronoi region of each cell are served by the cell. Each BS has $N_{t}$ transmit antennas and each user has $N_{r}$ receive antennas. We consider the ergodic spectral efficiency of each user as the main performance metric. Assuming that the BSs operate symmetrically, every user in the network has the same ergodic spectral efficiency. Therefore, we can select an arbitrary user in the network to evaluate the ergodic spectral efficiency of each user. We denote the corresponding user by user 1 and denote the serving BS of user 1 by BS 1. The remaining BSs except BS 1 are denoted as BS 2, BS 3, ⋯, using natural numbers greater than 1; and K−1 users served by BS 1, except user 1, is denoted by indices 2,⋯,K. It should be noted that the BS and user indices, except user 1 and BS 1, are arbitrarily assigned without any condition. Using Slivnyak’s theorem [16,30], we assume that user 1 is located at the origin without loss of generality. The random location of each BS is denoted as $d_{i}$, where *i* is the BS index in N, which follows a homogeneous PPP with density λ; thus, $d_{1}$ is the location of BS 1, which serves user 1. Therefore, the BS topology can be represented as $\Phi =\{d_{i},i\in N\}$. Each BS performs linear precoding for the SDM of *K* users.

Then, the received signal of user 1 is represented as

(16) $$\begin{array}{c} y_{1}=∥d_{1}{∥}^{-\frac{\alpha }{2}}\;H_{1,1}^{H}V_{1}s_{1}+\sum_{i=2}^{\infty }{∥d_{i}∥}^{-\frac{\alpha }{2}}\;H_{1,i}^{H}V_{i}s_{i}+z_{1}, \end{array}$$

where $H_{1,i}\in C^{N_{t}\times N_{r}}$ represents the channel matrix from BS *i* to user 1, $V_{i}=\left[v_{1}^{\left(i\right)},\cdots ,v_{K}^{\left(i\right)}\right]\in C^{N_{t}\times K}$ represents the precoding matrix of BS *i*, and $s_{i}$ represents an information symbol vector prepared for *K* users served by BS *i*. The entries of the channel matrix follow an i.i.d. complex Gaussian distribution with zero mean and unit variance, and $z_{1}$ is the complex additive Gaussian noise for user 1. It is assumed that the pathloss exponent α>2 and an equal amount of power is allocated to all users such that $E\left[s_{i}s_{i}^{H}\right]=\frac{P}{N_{t}}\cdot I_{N_{t}}$.

For simplicity, we omit the subscripts denoting BS 1 in (16), such that $∥d∥=∥d_{1}∥$, $H_{1}=H_{1,1}$, $V=\left[v_{1},\cdots ,v_{K}\right]=V_{1}$, and $s=s_{1}=\left[s_{1},\cdots ,s_{K}\right]$. Accordingly, (16) can be rewritten as follows:

(17) $$\begin{array}{cc} y_{1} & ={∥d∥}^{-\frac{\alpha }{2}}\;H_{1}^{H}v_{1}s_{1}+\sum_{k=2}^{K}{∥d∥}^{-\frac{\alpha }{2}}\;H_{1}^{H}v_{k}s_{k}+\sum_{i=2}^{\infty }{∥d_{i}∥}^{-\frac{\alpha }{2}}\;H_{1,i}^{H}V_{i}s_{i}+z_{1}. \end{array}$$

### 4.1. Channel Quantization and Limited Feedback

Each user determines their own receive combining vector and quantized CDI based on the QBC criterion, as described in Section 2. Subsequently, the index of the quantized CDI is reported to its serving BS, which obtains the quantized CDI of the user from the predefined codebook.

### 4.2. Zero-Forcing Beamforming

Zero-forcing beamforming is used to construct a linear precoding matrix. The ZFBF is a simple and efficient SDM strategy that allows each BS to simultaneously serve multiple users $K\leq N_{t}$. Let ${\hat{h}}_{k}$ be the quantized CDI of user *k* for k=1,⋯,K. Then, to obtain the ZFBF matrix, BS 1 takes the pseudo-inverse matrix of the aggregation of the quantized CDIs, such that [3]

(18) $$\begin{array}{cc} \; & \hat{H}=\left[{\hat{h}}_{1},...,{\hat{h}}_{K}\right],\;\;{\hat{H}}_{\mathrm{inv}}=\hat{H}{\left({\hat{H}}^{H}\hat{H}\right)}^{-1}. \end{array}$$

Each column $v_{j}$ of the precoding matrix V is the normalized *j*-th column vector of ${\hat{H}}_{\mathrm{inv}}$. Similarly, each BS *i* constructs its own ZFBF matrix $V_{i}$, based on the quantized CDIs received from its serving users. It should be noted that if we consider single-user communication per BS (K=1), the precoding matrix is reduced to the quantized CDI between the user and BS. For example, for BS 1 and user 1, we obtain

(19) $$\begin{array}{cc} \; & V={\hat{h}}_{1}. \end{array}$$

### 4.3. Performance Metrics

Let the QBC combining vector of user 1 be $\gamma_{1}$ and the effective channel of user 1 be $h_{1}^{e}=H_{1}\gamma_{1}$. Then, from (17), the signal-to-interference ratio (SIR) is expressed as [16]

(20) $$\begin{array}{cc} \mathrm{SIR}(B,K) & =\frac{\frac{P}{K}{∥d∥}^{-\alpha }\;{\left|{\left(h_{1}^{e}\right)}^{H}v_{1}\right|}^{2}}{\frac{P}{K}\sum_{k=2}^{K}{∥d∥}^{-\alpha }|{\left(h_{1}^{e}\right)}^{H}v_{k}{|}^{2}+\frac{P}{K}\sum_{i=2}^{\infty }∥d_{i}{∥}^{-\alpha }{∥\gamma_{1}^{H}H_{1,i}^{H}V_{i}∥}^{2}}\\ \; & =\frac{|{\left(h_{1}^{e}\right)}^{H}v_{1}{|}^{2}}{I_{U}\left(K\right)+I_{C}\left(K\right)}, \end{array}$$

where the intra-cell multiuser interference is defined as

(21) $$\begin{array}{cc} I_{U}\left(K\right) & ≜\sum_{k=2}^{K}{\left|{\left(h_{1}^{e}\right)}^{H}v_{k}\right|}^{2} \end{array}$$

and the inter-cell interference is

(22) $$\begin{array}{c} I_{C}\left(K\right)≜{∥d∥}^{\alpha }\sum_{i=2}^{\infty }∥d_{i}{∥}^{-\alpha }{∥\gamma_{1}^{H}H_{1,i}^{H}V_{i}∥}^{2}. \end{array}$$

The ergodic spectral efficiency is defined as [16]

(23) $$\begin{array}{c} R\left(B,K\right)=E[\log_{2}\left(1+\mathrm{SIR}\left(B,K\right)\right)]. \end{array}$$

This definition uses SIR instead of SINR, which means that it neglects the additive noise. The intuition behind this approximation is that the effect of additive noise is less important than interference in dense cellular networks [16,31].

Obviously, both the ergodic spectral efficiency R(B,K) and uplink resources required for feedback increase with the number of feedback bits *B*. Thus, the network service operator should consider a balance between the downlink and uplink resource usage. In this context, rather than R(B,K), we focus on the following net ergodic spectral efficiency [16]:

(24) $$\begin{array}{cc} \; & R_{\mathrm{Net}}\left(B,K\right)=R\left(B,K\right)-\frac{B}{T_{c}}=E\left[\log_{2}\left(1+\mathrm{SIR}\left(B,K\right)\right)\right]-\frac{B}{T_{c}}, \end{array}$$

where $T_{c}$ represents the channel coherence time, defined as the number of downlink channel uses that share the same channel realization. Accordingly, *B* bits are used to quantize $T_{c}$ consecutive downlink channel uses. Thus, the net spectral efficiency in (24) multiplied by $T_{c}$ is equivalent to the sum spectral efficiency over the $T_{c}$ channel uses ($T_{c}$ channel uses correspond to a single channel-coherent block) subtracted by the number of feedback bits. Thus, (24) measures the ergodic number of bits per downlink channel use, which can be transmitted with an arbitrarily small error, subtracted by the number of bits per channel use that is used to convey the feedback information. Therefore, it measures the effective data rate by considering the uplink resource dissipation.

Another important performance metric in this application is the optimal number of feedback bits that maximizes the ergodic net spectral efficiency. That is, the optimal number is defined as

(25) $$\begin{array}{cc} \; & B^{*}=\underset{B\in N}{\mathrm{argmax}}R_{\mathrm{Net}}\left(B,K\right). \end{array}$$

As discussed in [16,20], a large $T_{c}$ regime is considered in this study, in which the inaccuracy of the CSIT can equally degrade many downlink symbols. In this regime, the optimal number of feedback bits is relatively large. In the conventional quantization model, an explicit codebook C is needed to quantize CDI; thus, the simulation running time exponentially increases with *B*. Consequently, it is significantly difficult to determine $B^{*}$ in a large $T_{c}$ regime because the codebook size is considerably large near the optimal number $B^{*}$. Moreover, mathematical analysis is intractable owing to the complicated CDF (10) of the original quantization error. However, the proposed SCVQ model can be alternatively used to evaluate the optimal number of feedback bits by deriving the derivative of the spectral efficiency. To this end, it is useful to first relax the domain of *B* into a real number, rather than directly considering the original NP-hard problem. That is, we consider

(26) $$\begin{array}{cc} \; & B_{\mathrm{Real}}^{*}=\underset{B\in R}{\mathrm{argmax}}R_{\mathrm{Net}}\left(B,K\right), \end{array}$$

and $B^{*}$ is approximated as the integer closest to $B_{\mathrm{Real}}^{*}$. To obtain $B_{\mathrm{Real}}^{*}$, we consider the derivative of $R_{\mathrm{Net}}\left(B,K\right)$ (differentiating $R_{\mathrm{Net}}\left(B,K\right)$ becomes mathematically tractable with the proposed SCVQ model).

As an example, a special case of K=1 is considered for the evaluation of the optimal number of feedback bits. When K=1, multiuser interference does not exist (i.e., $I_{U}\left(1\right)=0$), and the precoding matrix V is reduced to ${\hat{h}}_{1}$, as expressed in (19), which is true for all the BSs and users in the network. Hence, SIR(B,1) is expressed as

(27) $$\begin{array}{c} \mathrm{SIR}\left(B,1\right)=\frac{|{\left(h_{1}^{e}\right)}^{H}{\hat{h}}_{1}{|}^{2}}{I_{C}\left(1\right)} \end{array}$$

where

(28) $$\begin{array}{c} I_{C}\left(1\right)={∥d∥}^{\alpha }\sum_{i=2}^{\infty }∥d_{i}{∥}^{-\alpha }∥d_{i}{∥}^{-\alpha }{∥\gamma_{1}^{H}H_{1,i}^{H}V_{i}∥}^{2}. \end{array}$$

From (23) and (27), we obtain

(29) $$\begin{array}{cc} R(B,1) & =E\left[\log_{2}\left(\frac{I_{C}\left(1\right)+{\left|{\left(h_{1}^{e}\right)}^{H}{\hat{h}}_{1}\right|}^{2}}{I_{C}\left(1\right)}\right)\right]. \end{array}$$

Furthermore, from (4) and (14), we have

(30) $$\begin{array}{cc} R(B,1) & =E[\log_{2}(I_{C}\left(1\right)+∥h_{1}^{e}{∥}^{2}\cos^{2}\theta_{k})]-E\left[\log_{2}I_{C}\left(1\right)\right]\\ \; & =E[\log_{2}(I_{C}\left(1\right)+∥h_{1}^{e}{∥}^{2}\left(1-\delta^{*}\beta_{N_{t}-N_{r},1}\right))]-E\left[\log_{2}I_{C}\left(1\right)\right]. \end{array}$$

Because the second term on the right-hand side of (30) is independent of *B*,

(31) $$\begin{array}{cc} \frac{\partial R(B,1)}{\partial B} & =\frac{1}{\log_{e}2}\frac{\partial \delta^{*}}{\partial B}\cdot E\left[\frac{-∥h_{1}^{e}{∥}^{2}\beta_{N_{t}-N_{r},1}}{I_{C}\left(1\right)+{∥h_{1}^{e}∥}^{2}\left(1-\delta^{*}\beta_{N_{t}-N_{r},1}\right)}\right]\\ \; & =\frac{\partial }{\partial B}\frac{2^{-\frac{B}{N_{t}-N_{r}}}}{\log_{e}2}{\left(\sum_{i=1}^{N_{r}}\frac{\Gamma (N_{t}-N_{r}+i-1)}{\Gamma \left(N_{t}-N_{r}\right)\Gamma \left(i\right)}\right)}^{-\frac{1}{N_{t}-N_{r}}}\cdot E\left[\frac{-∥h_{1}^{e}{∥}^{2}\beta_{N_{t}-N_{r},1}}{I_{C}\left(1\right)+{∥h_{1}^{e}∥}^{2}\left(1-\delta^{*}\beta_{N_{t}-N_{r},1}\right)}\right]\\ \; & =\frac{\delta^{*}}{N_{t}-N_{r}}\cdot E\left[\frac{∥h_{1}^{e}{∥}^{2}\beta_{N_{t}-N_{r},1}}{I_{C}\left(1\right)+{∥h_{1}^{e}∥}^{2}\left(1-\delta^{*}\beta_{N_{t}-N_{r},1}\right)}\right]. \end{array}$$

Subsequently, we can express

(32) $$\begin{array}{cc} \frac{\partial R_{\mathrm{Net}}\left(B,1\right)}{\partial B} & =\frac{\delta^{*}}{N_{t}-N_{r}}\cdot E\left[\frac{∥h_{1}^{e}{∥}^{2}\beta_{N_{t}-N_{r},1}}{I_{C}\left(1\right)+{∥h_{1}^{e}∥}^{2}\left(1-\delta^{*}\beta_{N_{t}-N_{r},1}\right)}\right]-\frac{1}{T_{c}}. \end{array}$$

Because $\delta^{*}={\left(2^{B}\sum_{i=1}^{N_{r}}\frac{\Gamma (N_{t}-N_{r}+i-1)}{\Gamma \left(N_{t}-N_{r}\right)\Gamma \left(i\right)}\right)}^{-\frac{1}{N_{t}-N_{r}}}$ is a monotonically decreasing function of *B*, $\frac{\partial R(B,1)}{\partial B}$ monotonically decreases with increasing *B*. Thus, $B_{\mathrm{Real}}^{*}$ is determined as a unique real number that satisfies $\frac{\partial R_{\mathrm{Net}}\left(B,1\right)}{\partial B}=0$.

### 4.4. Simulation Results

In this section, simulation results are used to verify the validity of the proposed SCVQ model. For the BS topology, a homogeneous PPP with a density of $\lambda =10^{-5}/\pi$ is used, and the pathloss exponent is fixed at α=4. For simplicity, the network area is set to a circle with radius 5000 [m]. Theoretically, the network size of a homogeneous PPP must be infinity, but this is not feasible in practice. Thus, we assume a sufficiently large network area. Expected rates are simulated based on the Monte Carlo method, and the number of trials to obtain the expected values is set to be sufficiently large to obtain reliable results.

**Figure 1:**
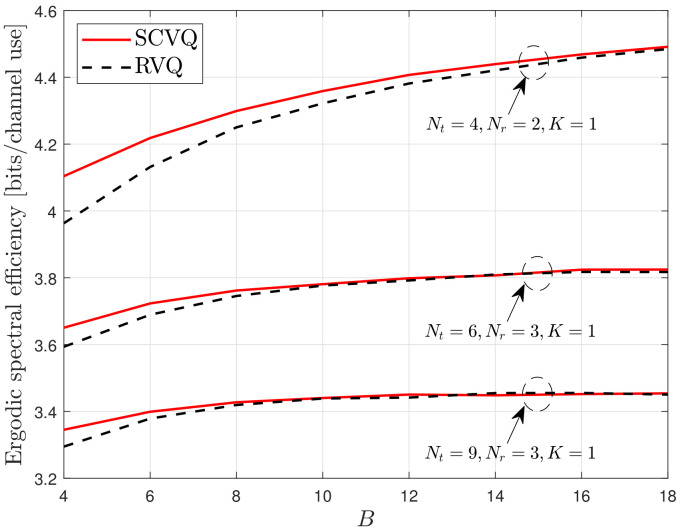
Verification of the proposed SCVQ.

**Figure 2:**
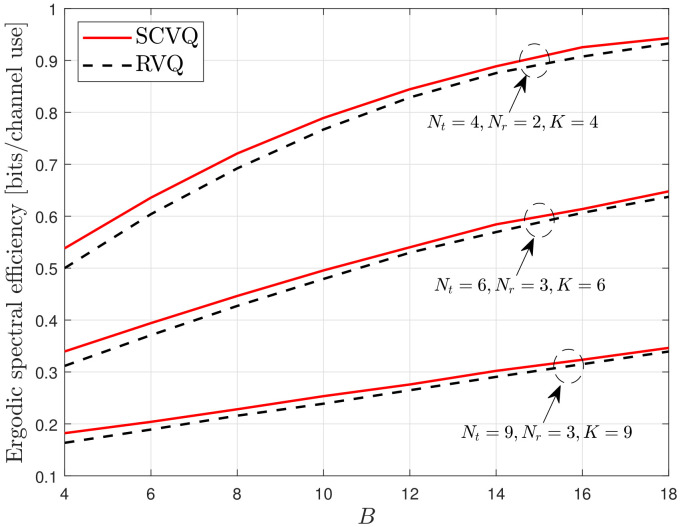
Verification of the proposed SCVQ.

**Figure 3:**
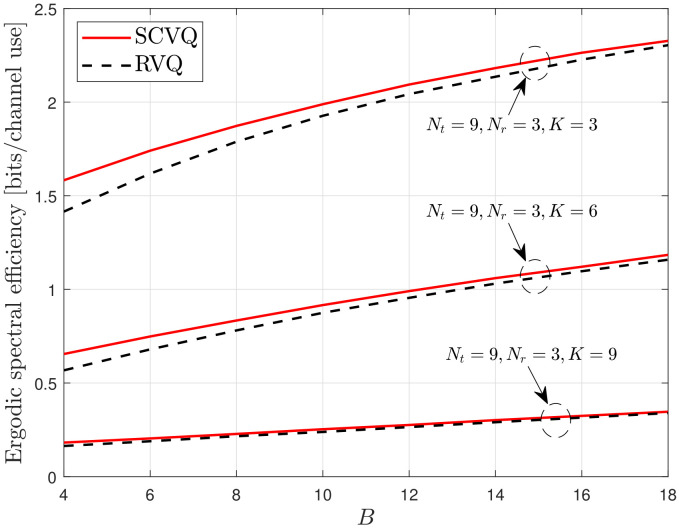
Verification of the proposed SCVQ.

First, we verify the accuracy of the proposed spherical-cap approximation, which is described in (13) and (14). As expressed in (11), the proposed SCVQ provides an upper bound for the CDF of the quantization error $\sin^{2}\theta$ with respect to the quantization codebook construction. Thus, the proposed SCVQ naturally provides upper-bound ergodic spectral efficiency compared with any explicit codebook. Moreover, RVQ yields a lower-bound performance compared with a well-designed explicit codebook because it provides the ensemble average with respect to random codebooks [2]. Figure 1, Figure 2 and Figure 3 demonstrate the gap between the lower- and upper-bound performances achieved by the RVQ and the proposed SCVQ, respectively. As expected, the corresponding spectral efficiency gap is generally small and decreases with an increasing number of feedback bits. This is because the intersection between two arbitrary Voronoi regions of codewords ($\left\{X_{i}\leq x\right\}\cap \left\{X_{j}\leq x\right\}$) decreases with increasing *B* when the union bound in (11) is applied. Because the spectral efficiency achieved with an optimally designed realistic codebook, such as the codebook in [7], is between those achieved with RVQ and SCVQ, it is reasonable to conclude that the proposed SCVQ provides a close approximation to a well-designed explicit codebook in dense cellular networks.

**Figure 4:**
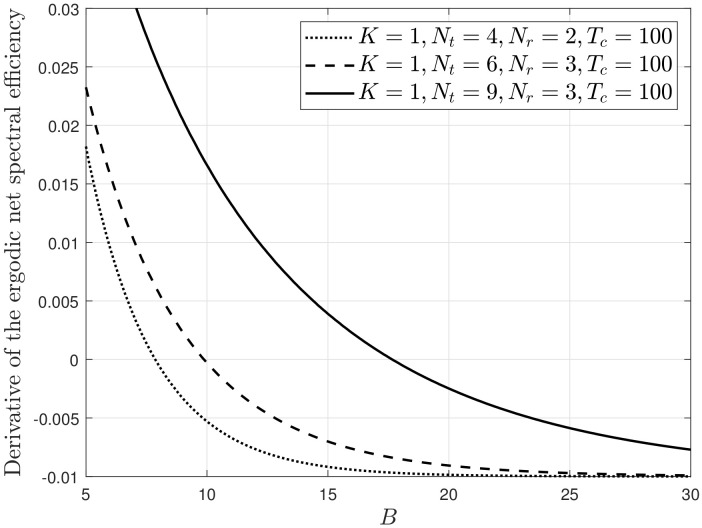
$\frac{\partial R_{\mathrm{Net}}\left(B,1\right)}{\partial B}$ vs. *B*.

**Figure 5:**
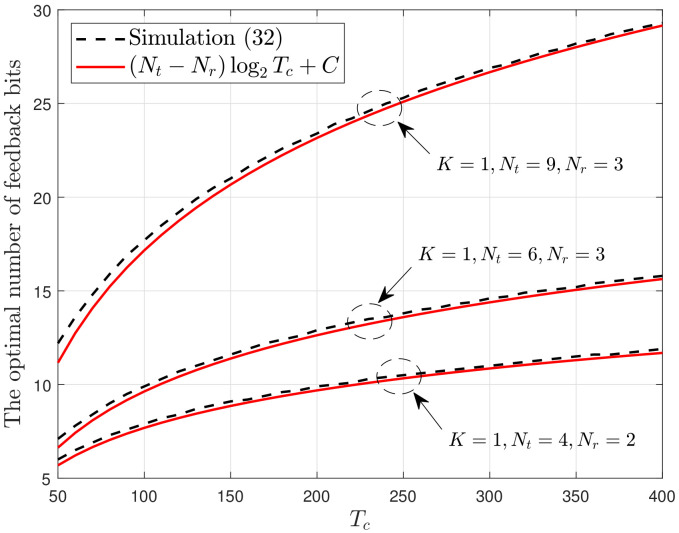
$B_{\mathrm{Real}}^{*}$ vs. $T_{c}$.

Figure 4 depicts the derivative of the net spectral efficiency $\frac{\partial R_{\mathrm{Net}}\left(B,1\right)}{\partial B}$ with respect to *B* when the channel coherence time is fixed at 100. As specified in (32), $\frac{\partial R_{\mathrm{Net}}\left(B,1\right)}{\partial B}$ is a monotonically decreasing function of *B*. The unique zero-crossing point of each curve provides the optimal number of feedback bits. Figure 5 depicts $B_{\mathrm{Real}}^{*}$ as a function of $T_{c}$, which is obtained by determining the zero-crossing point. When $N_{r}=1$, $B_{\mathrm{Real}}^{*}$ is scaled to $\left(N_{t}-1\right)\log_{2}T_{c}$ [16,20]. When $N_{r}>1$ and QBC is used, $B_{\mathrm{Real}}^{*}$ is scaled at a rate of $\left(N_{t}-N_{r}\right)\log_{2}T_{c}$, as shown in Figure 5, implying that the scaling of the optimal number is reduced if we implement multiple receive antennas to reduce the quantization error. In Figure 5, the constant *C* is numerically determined for a direct comparison and it is different for different values of *K*, $N_{t}$ and $N_{r}$. The aggregated number of feedback bits in each cell is then scaled at a rate of $K\left(N_{t}-N_{r}\right)\log_{2}T_{c}$, which is equal to the rate obtained when we use block diagonalization rather than antenna combining at the receiver end [32].

### 4.5. Discussion

In this section, the proposed SCVQ is verified in a dense cellular network modeled by a random PPP. The SCVQ is an analytical model that approximates the quantization error achieved by a well-designed codebook (a well-designed codebook indicates any explicit codebook that performs better than RVQ). Moreover, because the proposed SCVQ was derived using a probability upper bound (the union bound), the probability that the quantization error is smaller than a fixed value is always greater with the proposed SCVQ than with any explicit codebook. Thus, the downlink rate with the proposed SCVQ must achieve upper-bound performance in terms of codebook construction, as verified in Figure 1, Figure 2 and Figure 3. Furthermore, because the RVQ codebook is randomly generated for each channel realization independent of the channel fading, there always exists at least one fixed codebook that performs better than the RVQ, which indicates that the RVQ provides lower-bound performance in terms of codebook construction [9]. Thus, in Figure 1, Figure 2 and Figure 3, the rate gap between the cases with SCVQ and RVQ indicates the gap between the upper- and lower-bound rates such that the rate achieved by a well-designed realistic codebook will lie between them. Consequently, the small rate gaps in Figure 1, Figure 2 and Figure 3 imply the accuracy of the proposed spherical-cap approximation.

In conclusion, the proposed SCVQ was verified to be useful for approximating the quantization process using an explicit codebook. Because SCVQ significantly simplifies the quantization process, it is useful for mathematical analysis with limited feedback. The results in Figure 4 and Figure 5 demonstrate this advantage.

## 5. Conclusions

In this study, we introduced a generalized version of the SCVQ that is applicable to QBC. The proposed SCVQ model closely emulates the quantization error induced by the finite-rate quantization of CDI with QBC by replacing the quantization error with a beta random variable multiplied by a constant. Because the beta random variable is generated independently of the number of feedback bits, the proposed model provides relatively low simulation complexity invariant with the number of feedback bits. Moreover, the proposed model enables mathematical analysis, which is difficult to conduct using the conventional explicit-codebook-based approach. The advantage of the proposed SCVQ model was verified in a dense cellular network in which each BS performed downlink SDM based on limited feedback. The simulation results demonstrated the accuracy of the proposed spherical-cap approximation. With the proposed SCVQ, the quantization process can be significantly simplified such that performance analysis, which is extremely difficult, can become mathematically tractable. In the cellular network, we simulated the downlink rate of a limited-feedback-based MIMO system and demonstrated that the simulation results are consistent with the analysis results, which were obtained assuming the proposed SCVQ. The corresponding results verified the advantages of the proposed SCVQ in terms of mathematical analysis.

## Figures and Tables

**Table 1:** Related work on codebook design and spherical-cap approximation.

	Previous Studies	Relative Difficulty in Theoretical Analysis	Ergodic Rate Compared with a Well-Designed Codebook
Fixed codebook	[7,8]	high	-
RVQ	[9,10,11,13,14]	low	lower-bound approximation
SCVQ	[12,15,16,17,18,19,20,21,22,23]	very low	upper-bound approximation

## Data Availability

Not applicable.
